# ASO Author Reflections: Impact of an Online Risk Calculator for Sentinel Node Positivity on Management of Patients with T1 and T2 Melanomas

**DOI:** 10.1245/s10434-024-15590-5

**Published:** 2024-06-14

**Authors:** Alec A. Winder, Andrew J. Spillane, Andrea L. Smith

**Affiliations:** 1 Melanoma Institute Australia, The University of Sydney, Sydney, NSW Australia; 2 Faculty Medicine and Health, The University of Sydney, Sydney, NSW Australia; 3 Royal North Shore Hospital, Sydney, NSW Australia; 4 Mater Hospital, Sydney, NSW Australia; 5 Faculty of Medicine, Health and Human Sciences, Macquarie University, Sydney, NSW Australia; 6 The Daffodil Centre, University of Sydney, A Joint Venture with Cancer Council NSW, Sydney, NSW Australia

## Past

Approximately 70–80% of primary melanomas are thin melanomas (≤1 mm). Most can be treated with wide local excision alone with a 10-year survival rate of 85–90%.^1^ A subset of patients with thin melanoma will, however, develop regional and distant metastases. These patients account for approximately 25% of melanoma deaths overall.^2^ While sentinel lymph node (SLN) positivity is a predictor of worse prognosis in thin melanoma patients, the vast majority (94.4%) of cases are negative.^3^ Predicting SLN positivity for thin melanoma patients remains difficult.

To better stratify the risk for all individual melanoma patients having SLN metastasis the Melanoma Institute Australia (MIA) developed a risk prediction calculator that included patient age, Breslow thickness, mitoses, ulceration, lymphovascular invasion and tumour subtype which was made available to the public in August 2020.^4^ We sought to evaluate the effects of the risk calculators on clinical practice on SLN biopsy (SLNB) for T1–2 melanomas after 1 year of implementation.

Present

Our study^5^ of the host institution’s experience showed the introduction of the MIA calculator was associated with an increased rate of SLNB for patients with “calculator defined higher risk” T1a melanomas with indications of an increase in SLN metastasis detection for this group. There was also a trend towards patients with higher risk T1b melanomas to undergo more SLNBs and those with lower risk T2a melanomas to undergo less. Overall rates of SLNB and SLN positivity were unchanged.

Due to relatively small group numbers results should be interpreted with caution. The decision making to perform SLNB is complex and likely impacted by the calculator, current guidelines and individual patient risk threshold, medical fitness and practical issues such as primary tumour location and lymphatic drainage patterns.

Future

Larger patient numbers and regional recurrence data will be needed to confirm the MIA calculator’s true effect on clinical practice. Currently SLNB still remains an important tool for staging and providing prognostic information for melanoma patients. In the future, better biomarkers, or prognostic gene expression profiles (GEP) may provide enough information for prognostication and treatment planning to obviate the need for SLNB. However, GEP testing is expensive and may be of greatest benefit for patients with thin to intermediate thickness melanomas with a negative SLNB. Currently the MIA calculator is freely available and provides more accurate prediction of SLN metastasis than other calculators and is more nuanced than guideline recommendations alone.^4^
